# Supplementary material for: A Humanized Yeast Phenomic Model of Deoxycytidine Kinase to Predict Genetic Buffering of Nucleoside Analog Cytotoxicity
Source: Genes (Basel). 2019 Sep 30;10(10):770. doi: 10.3390/genes10100770 (PMC6826991; doi:10.3390/genes10100770)
Supplement: Supplementary file 1 [file genes-10-00770-s001.zip › Additional_Files/Additional_File7_GOTermHeatmaps.zip!/Additional_File7_GOTermHeatmaps/spliceosomal_complex.pdf]

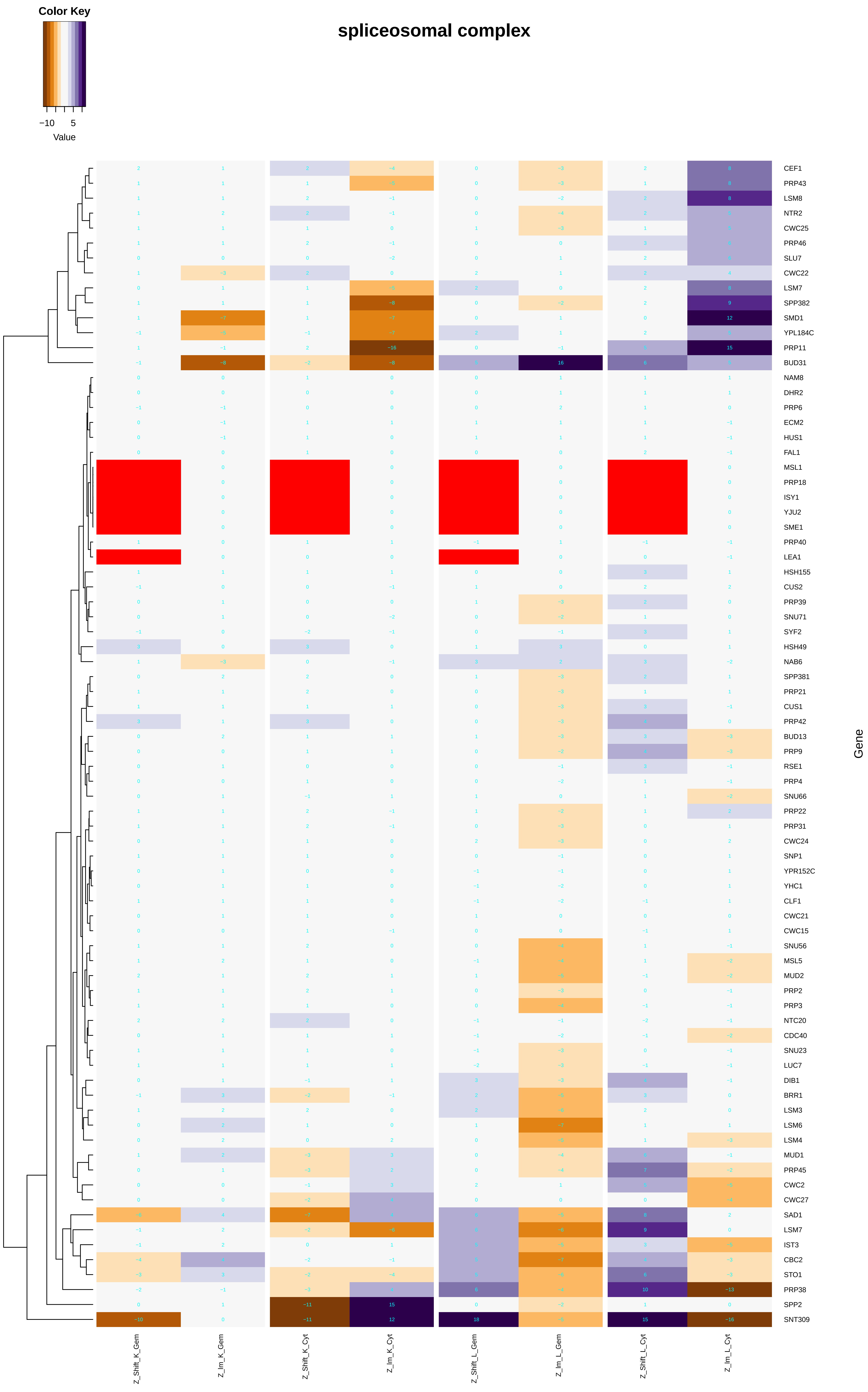

spliceosomal complex
Color Key
−10
5
Value
Gene
CEF1
PRP43
LSM8
NTR2
CWC25
PRP46
SLU7
CWC22
LSM7
SPP382
SMD1
YPL184C
PRP11
BUD31
NAM8
DHR2
PRP6
ECM2
HUS1
FAL1
MSL1
PRP18
ISY1
YJU2
SME1
PRP40
LEA1
HSH155
CUS2
PRP39
SNU71
SYF2
HSH49
NAB6
SPP381
PRP21
CUS1
PRP42
BUD13
PRP9
RSE1
PRP4
SNU66
PRP22
PRP31
CWC24
SNP1
YPR152C
YHC1
CLF1
CWC21
CWC15
SNU56
MSL5
MUD2
PRP2
PRP3
NTC20
CDC40
SNU23
LUC7
DIB1
BRR1
LSM3
LSM6
LSM4
MUD1
PRP45
CWC2
CWC27
SAD1
LSM7
IST3
CBC2
STO1
PRP38
SPP2
SNT309
Z_Shift_K_Gem
Z_Im_K_Gem
Z_Shift_K_Cyt
Z_Im_K_Cyt
Z_Shift_L_Gem
Z_Im_L_Gem
Z_Shift_L_Cyt
Z_Im_L_Cyt

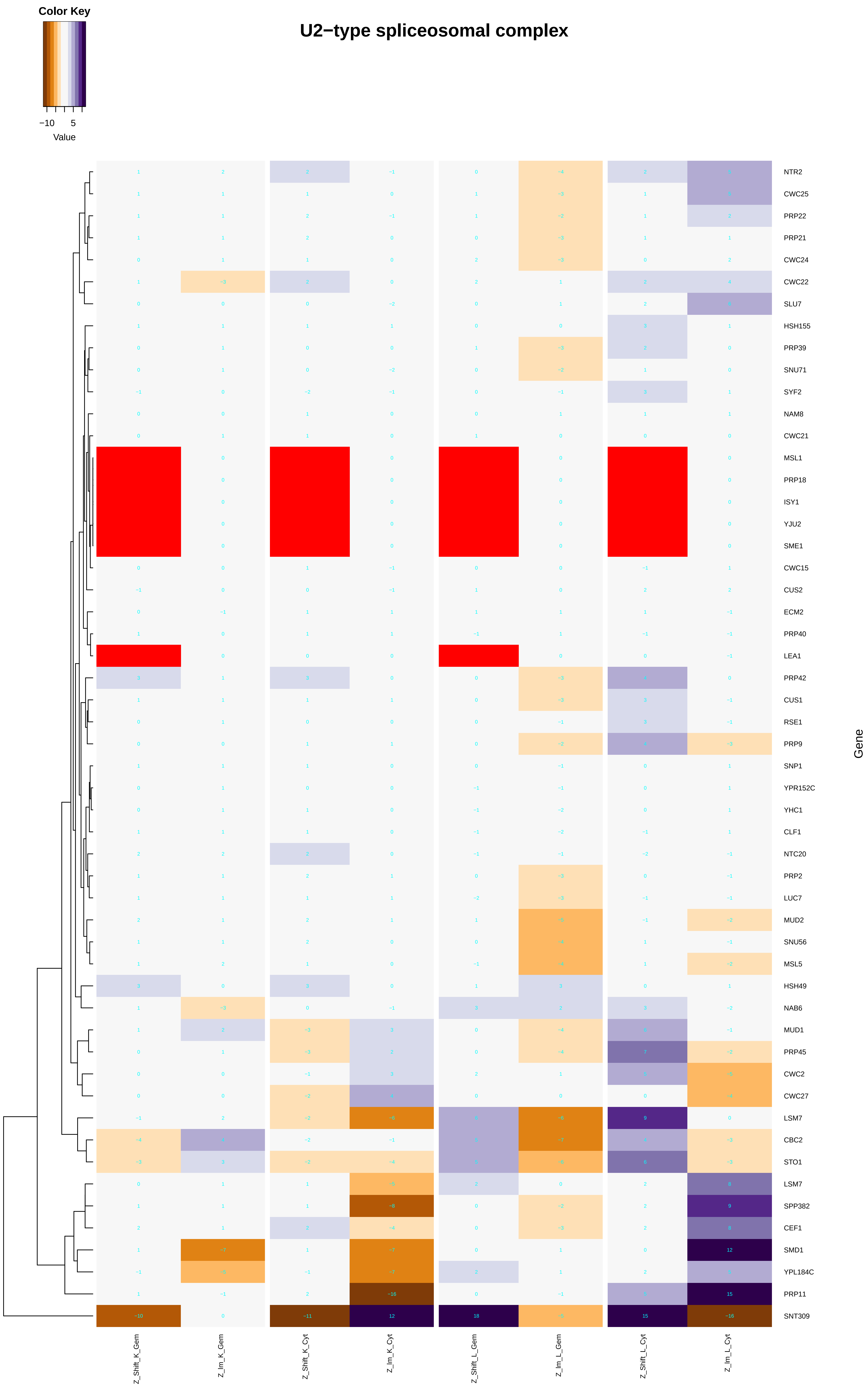

Color Key
−10
5
Value
U2−type spliceosomal complex
NTR2
CWC25
PRP22
PRP21
CWC24
CWC22
SLU7
HSH155
PRP39
SNU71
SYF2
NAM8
CWC21
MSL1
PRP18
ISY1
YJU2
SME1
CWC15
CUS2
ECM2
PRP40
LEA1
PRP42
CUS1
RSE1
PRP9
SNP1
YPR152C
YHC1
CLF1
NTC20
PRP2
LUC7
MUD2
SNU56
MSL5
HSH49
NAB6
MUD1
PRP45
CWC2
CWC27
LSM7
CBC2
STO1
LSM7
SPP382
CEF1
SMD1
YPL184C
PRP11
SNT309
Gene
Z_Shift_K_Gem
Z_Im_K_Gem
Z_Shift_K_Cyt
Z_Im_K_Cyt
Z_Shift_L_Gem
Z_Im_L_Gem
Z_Shift_L_Cyt
Z_Im_L_Cyt

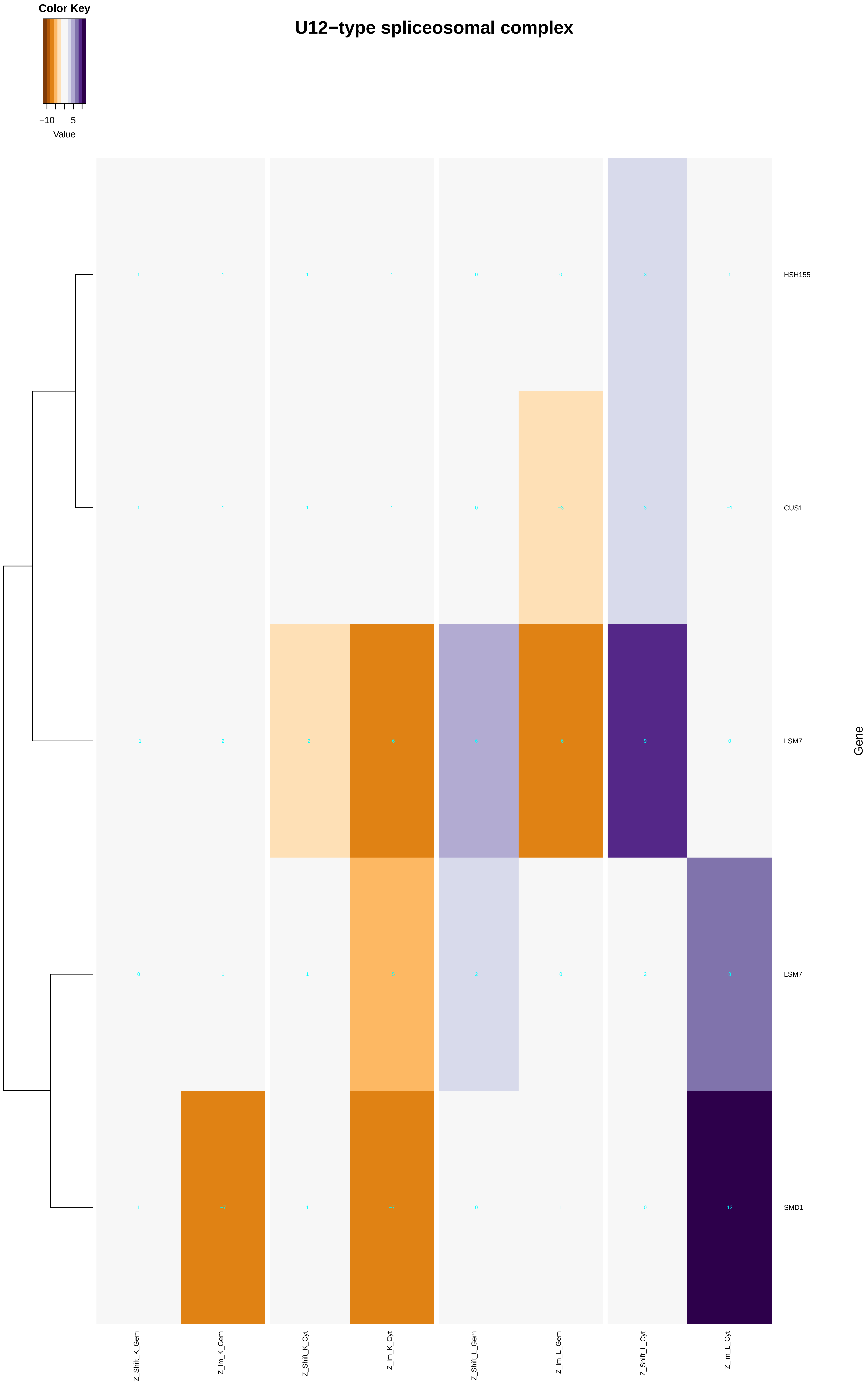

Color Key
−10
5
Value
U12−type spliceosomal complex
1 1 1 1 0 0 3 1
HSH155
1 1 1 1 0 −3 3 −1
CUS1
−1 2 −2 −6 6 −6 9 0
LSM7
0 1 1 −5 2 0 2 8
LSM7
1 −7 1 −7 0 1 0 12
SMD1
Gene
Z_Shift_K_Gem
Z_Im_K_Gem
Z_Shift_K_Cyt
Z_Im_K_Cyt
Z_Shift_L_Gem
Z_Im_L_Gem
Z_Shift_L_Cyt
Z_Im_L_Cyt

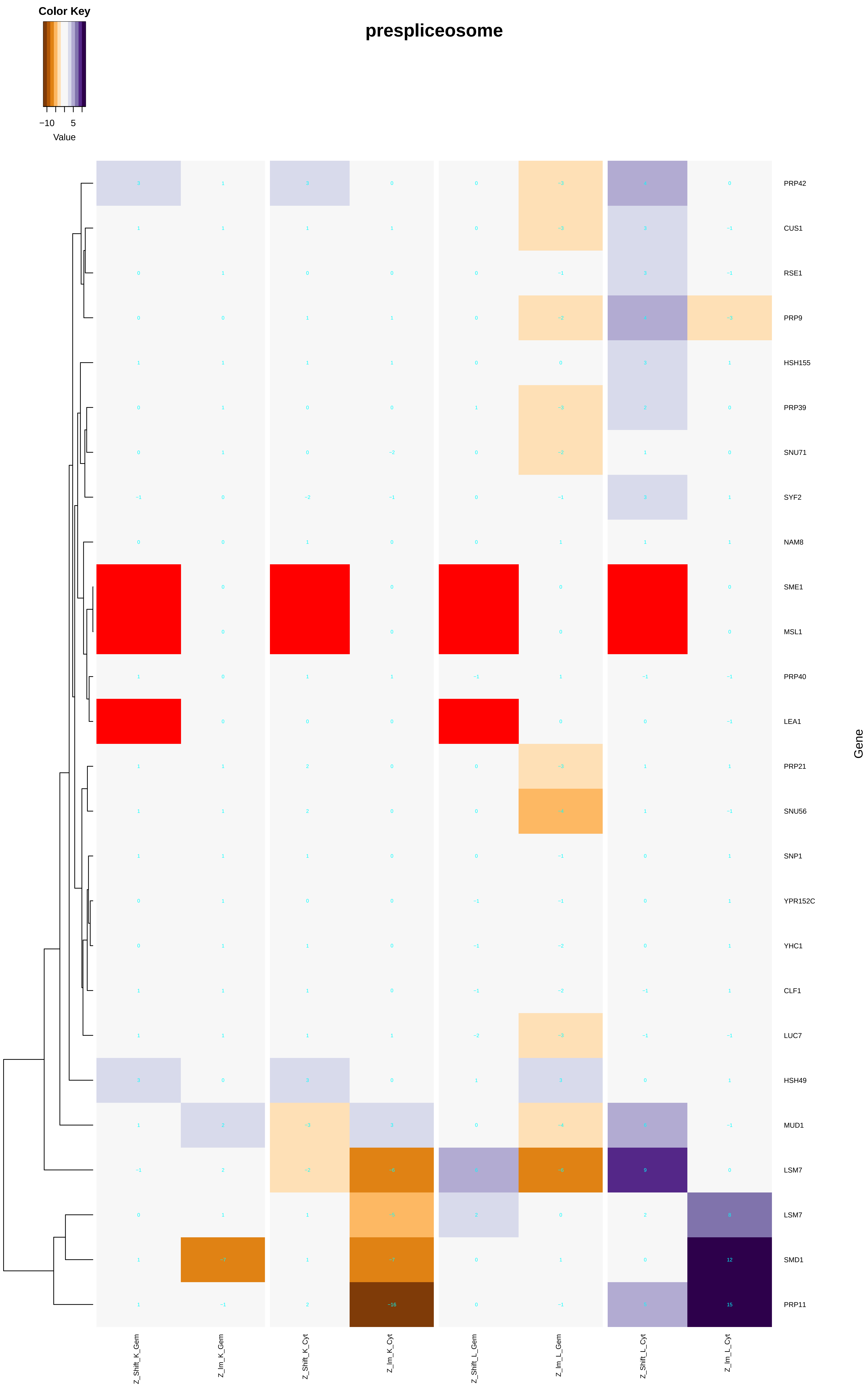
Color Key
−10
5
Value
prespliceosome
PRP42
CUS1
RSE1
PRP9
HSH155
PRP39
SNU71
SYF2
NAM8
SME1
MSL1
PRP40
LEA1
PRP21
SNU56
SNP1
YPR152C
YHC1
CLF1
LUC7
HSH49
MUD1
LSM7
LSM7
SMD1
PRP11
Gene
Z_Shift_K_Gem
Z_Im_K_Gem
Z_Shift_K_Cyt
Z_Im_K_Cyt
Z_Shift_L_Gem
Z_Im_L_Gem
Z_Shift_L_Cyt
Z_Im_L_Cyt

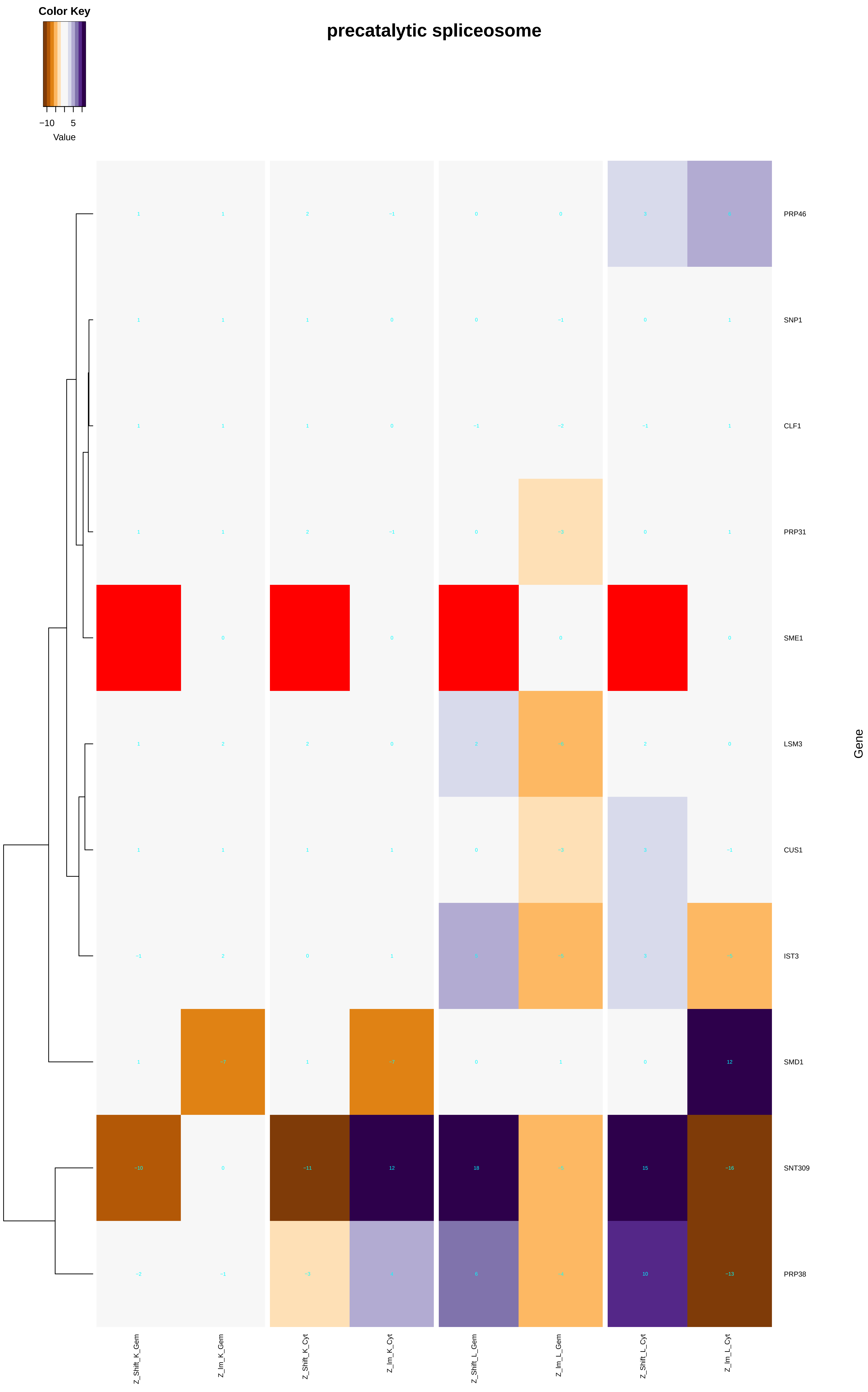

precatalytic spliceosome
Color Key
−10
5
Value
PRP46
SNP1
CLF1
PRP31
SME1
LSM3
CUS1
IST3
SMD1
SNT309
PRP38
Gene
Z_Shift_K_Gem
Z_Im_K_Gem
Z_Shift_K_Cyt
Z_Im_K_Cyt
Z_Shift_L_Gem
Z_Im_L_Gem
Z_Shift_L_Cyt
Z_Im_L_Cyt

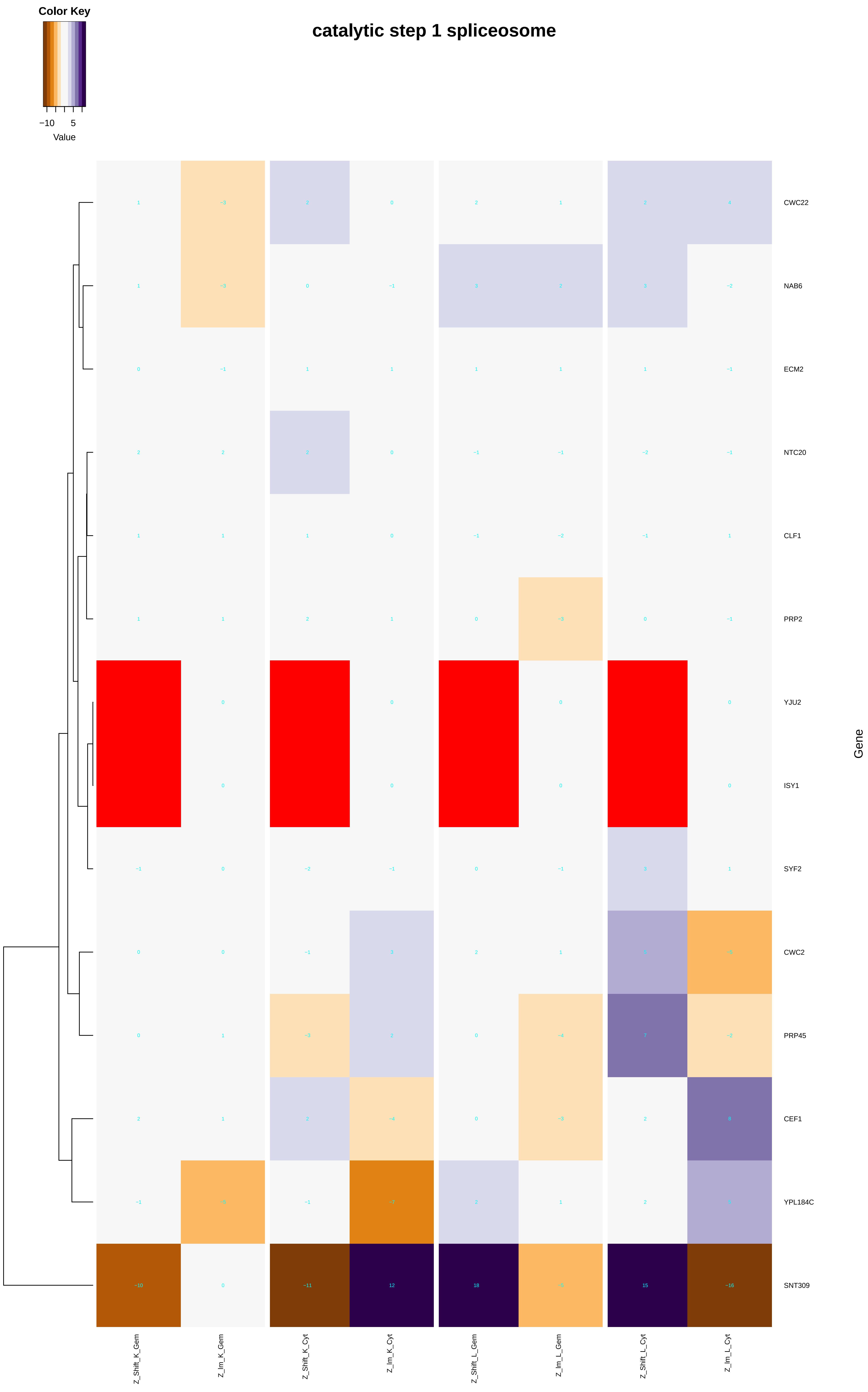
Color Key
−10
5
Value
catalytic step 1 spliceosome
CWC22
NAB6
ECM2
NTC20
CLF1
PRP2
YJU2
ISY1
SYF2
CWC2
PRP45
CEF1
YPL184C
SNT309
Gene
Z_Shift_K_Gem
Z_Im_K_Gem
Z_Shift_K_Cyt
Z_Im_K_Cyt
Z_Shift_L_Gem
Z_Im_L_Gem
Z_Shift_L_Cyt
Z_Im_L_Cyt

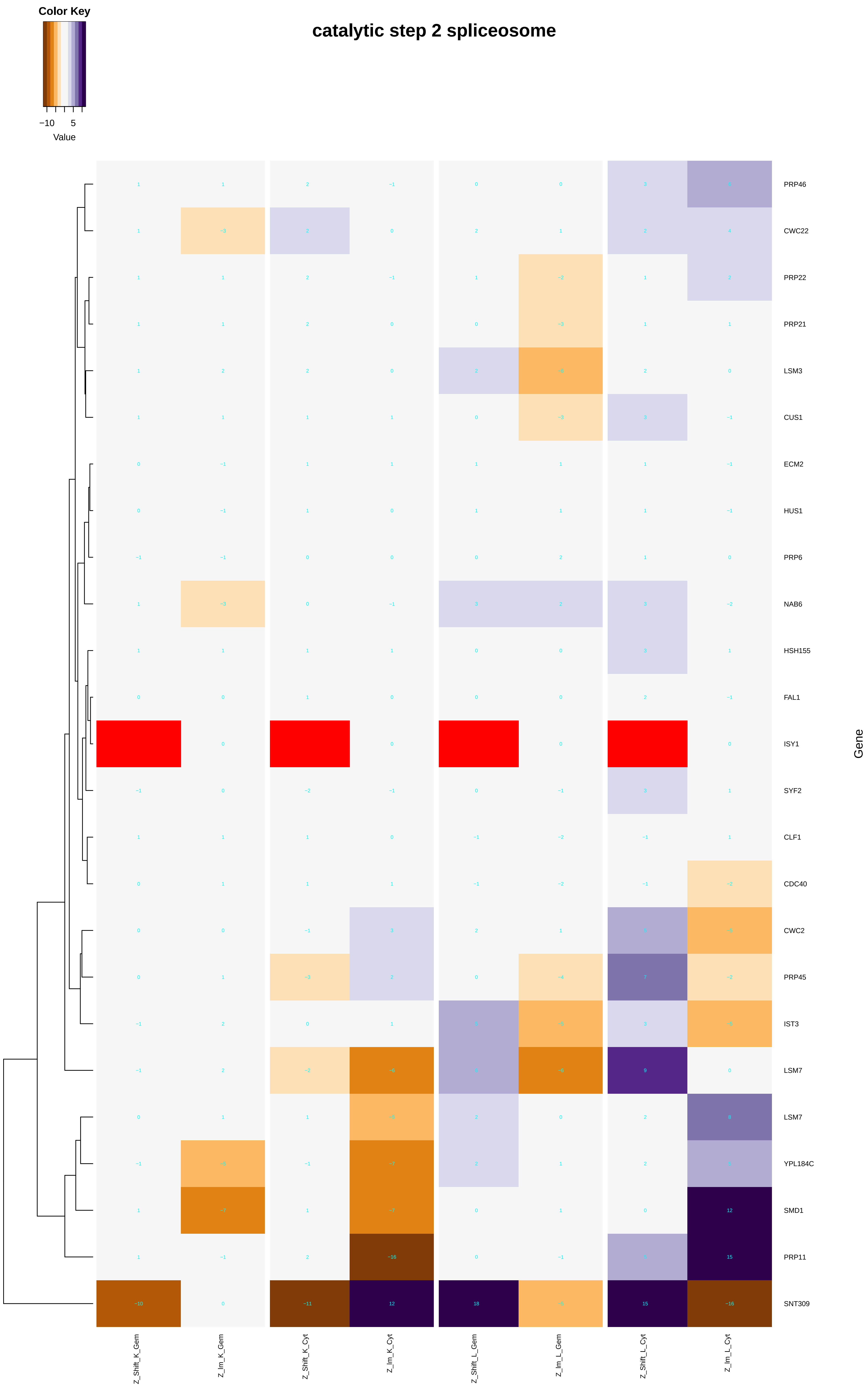
Color Key
−10
5
Value
catalytic step 2 spliceosome
PRP46
CWC22
PRP22
PRP21
LSM3
CUS1
ECM2
HUS1
PRP6
NAB6
HSH155
FAL1
ISY1
SYF2
CLF1
CDC40
CWC2
PRP45
IST3
LSM7
LSM7
YPL184C
SMD1
PRP11
SNT309
Gene
Z_Shift_K_Gem
Z_Im_K_Gem
Z_Shift_K_Cyt
Z_Im_K_Cyt
Z_Shift_L_Gem
Z_Im_L_Gem
Z_Shift_L_Cyt
Z_Im_L_Cyt

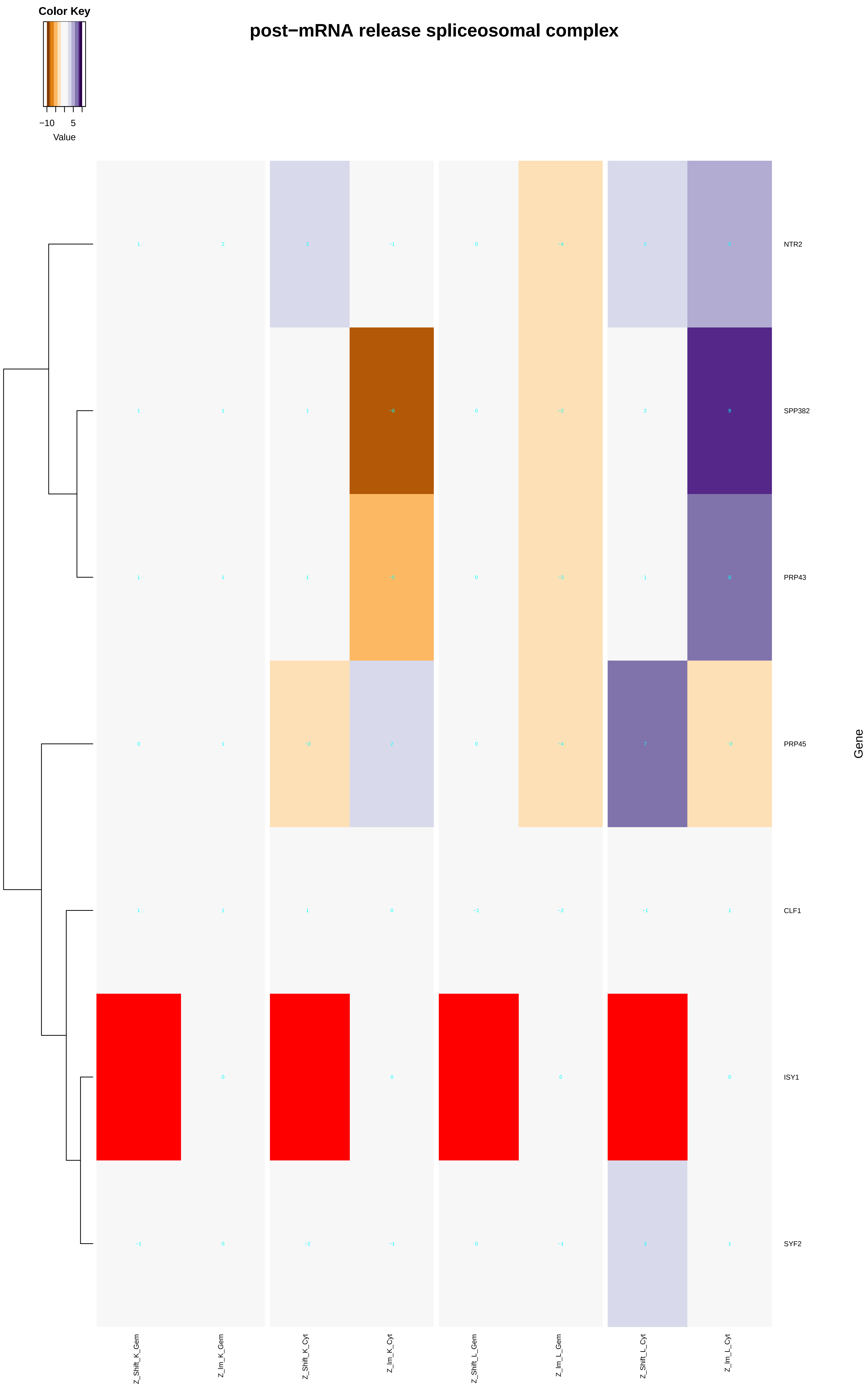
Color Key
−10
5
Value
post−mRNA release spliceosomal complex
NTR2
SPP382
PRP43
PRP45
CLF1
ISY1
SYF2
Gene
Z_Shift_K_Gem
Z_Im_K_Gem
Z_Shift_K_Cyt
Z_Im_K_Cyt
Z_Shift_L_Gem
Z_Im_L_Gem
Z_Shift_L_Cyt
Z_Im_L_Cyt

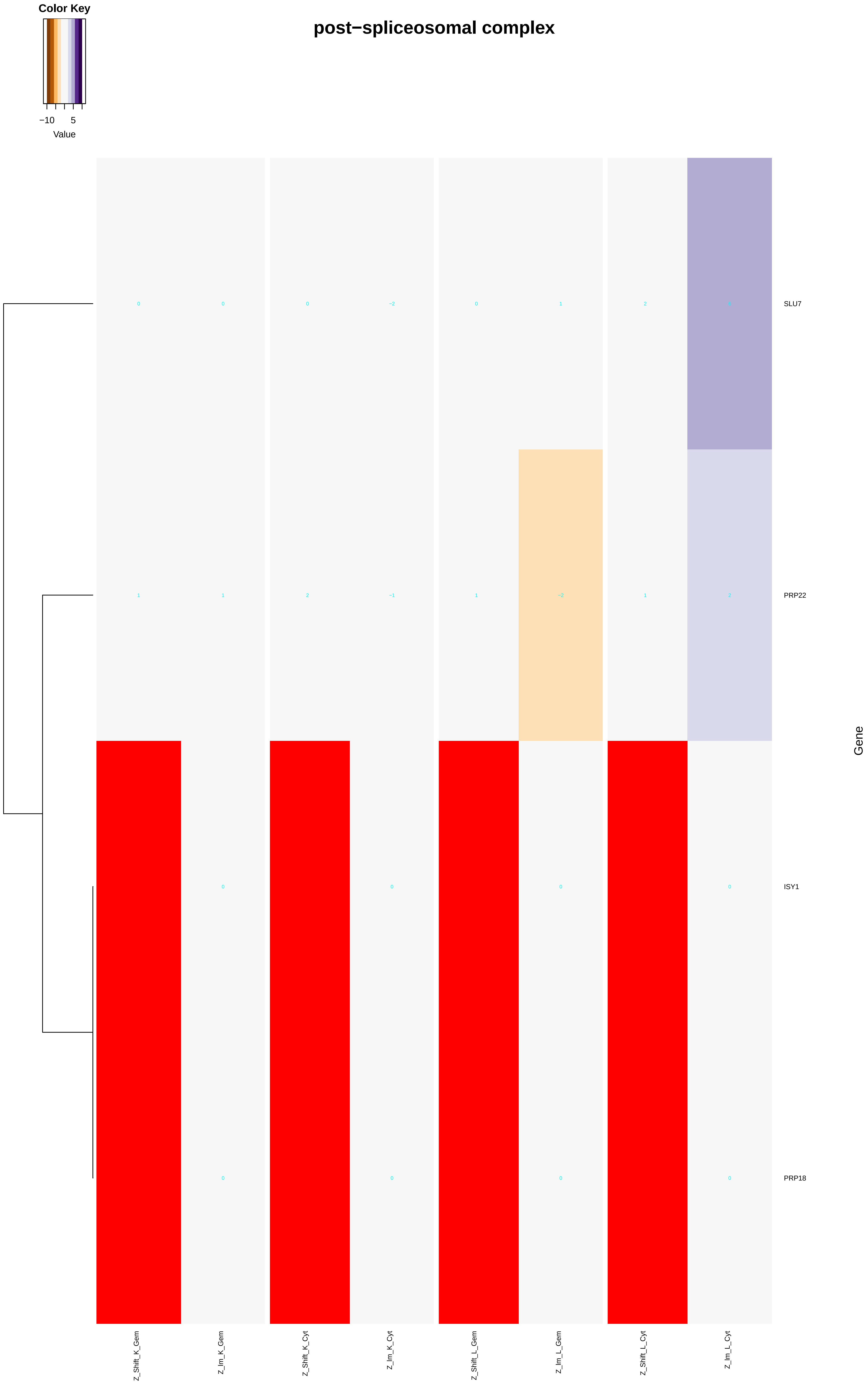
Color Key
−10
5
Value
post−spliceosomal complex
SLU7
PRP22
ISY1
PRP18
Gene
Z_Shift_K_Gem
Z_Im_K_Gem
Z_Shift_K_Cyt
Z_Im_K_Cyt
Z_Shift_L_Gem
Z_Im_L_Gem
Z_Shift_L_Cyt
Z_Im_L_Cyt

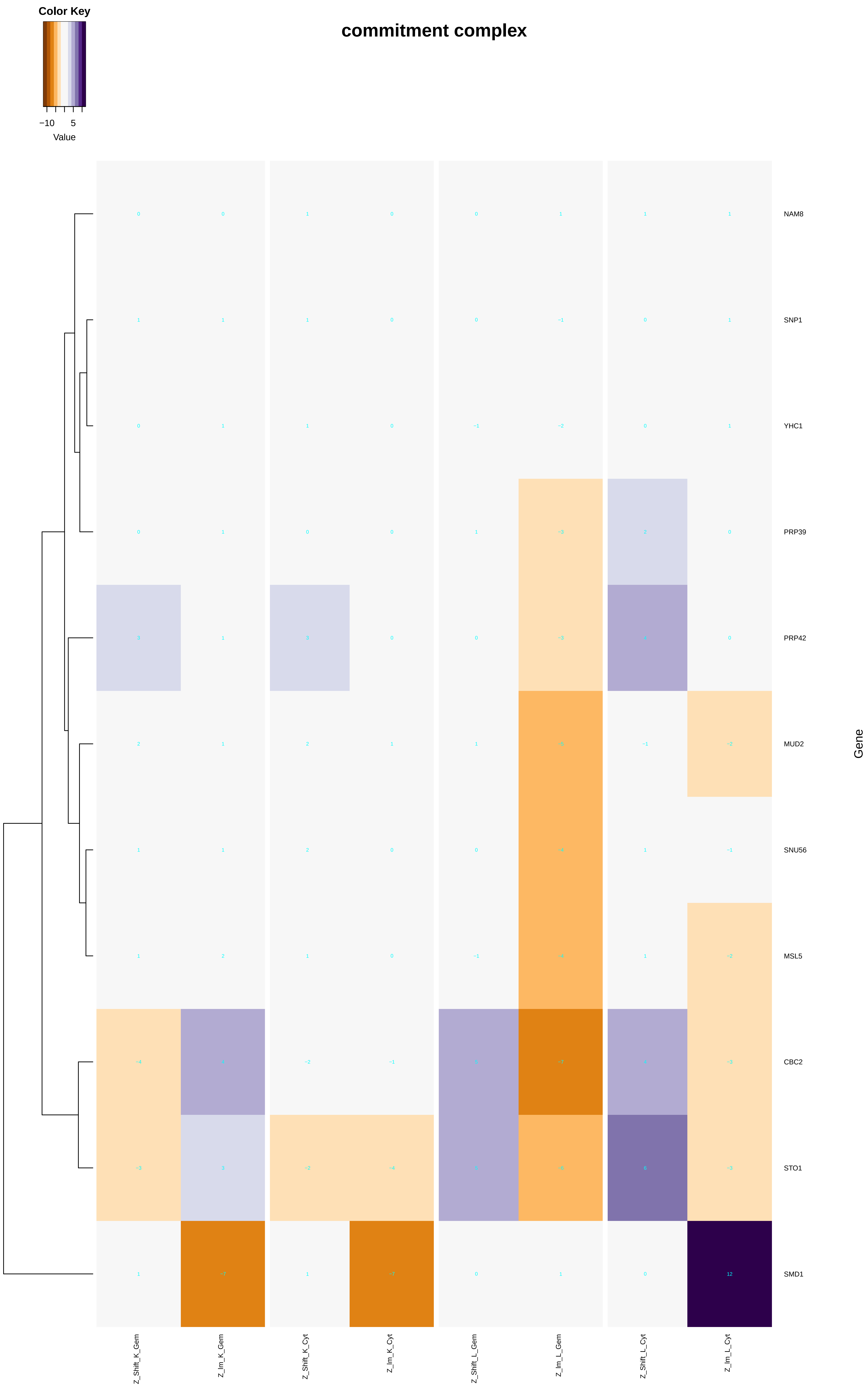
Color Key
−10
5
Value
commitment complex
NAM8
SNP1
YHC1
PRP39
PRP42
MUD2
SNU56
MSL5
CBC2
STO1
SMD1
Gene
Z_Shift_K_Gem
Z_Im_K_Gem
Z_Shift_K_Cyt
Z_Im_K_Cyt
Z_Shift_L_Gem
Z_Im_L_Gem
Z_Shift_L_Cyt
Z_Im_L_Cyt

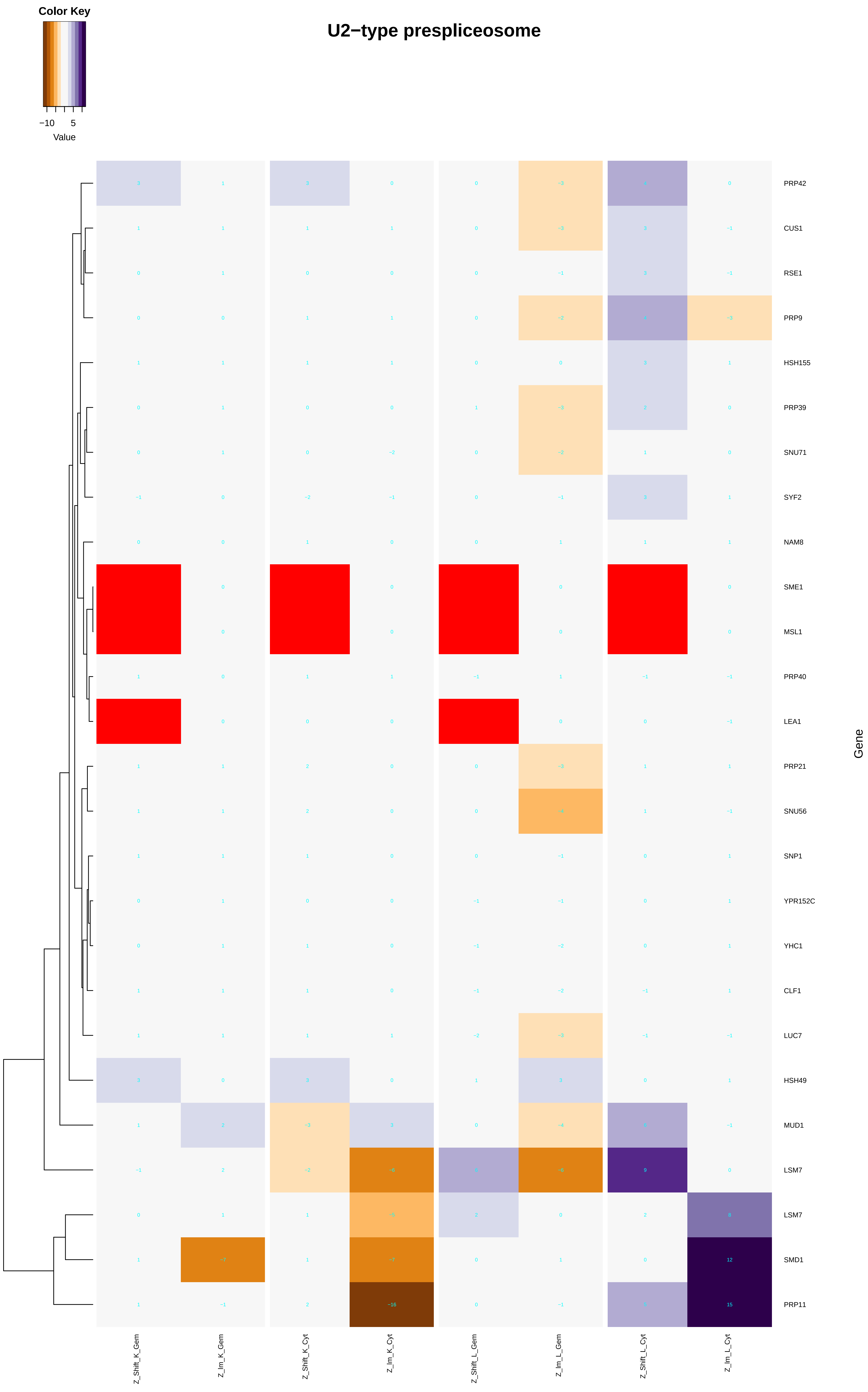
Color Key
−10
5
Value
U2−type prespliceosome
PRP42
CUS1
RSE1
PRP9
HSH155
PRP39
SNU71
SYF2
NAM8
SME1
MSL1
PRP40
LEA1
PRP21
SNU56
SNP1
YPR152C
YHC1
CLF1
LUC7
HSH49
MUD1
LSM7
LSM7
SMD1
PRP11
Gene
Z_Shift_K_Gem
Z_Im_K_Gem
Z_Shift_K_Cyt
Z_Im_K_Cyt
Z_Shift_L_Gem
Z_Im_L_Gem
Z_Shift_L_Cyt
Z_Im_L_Cyt

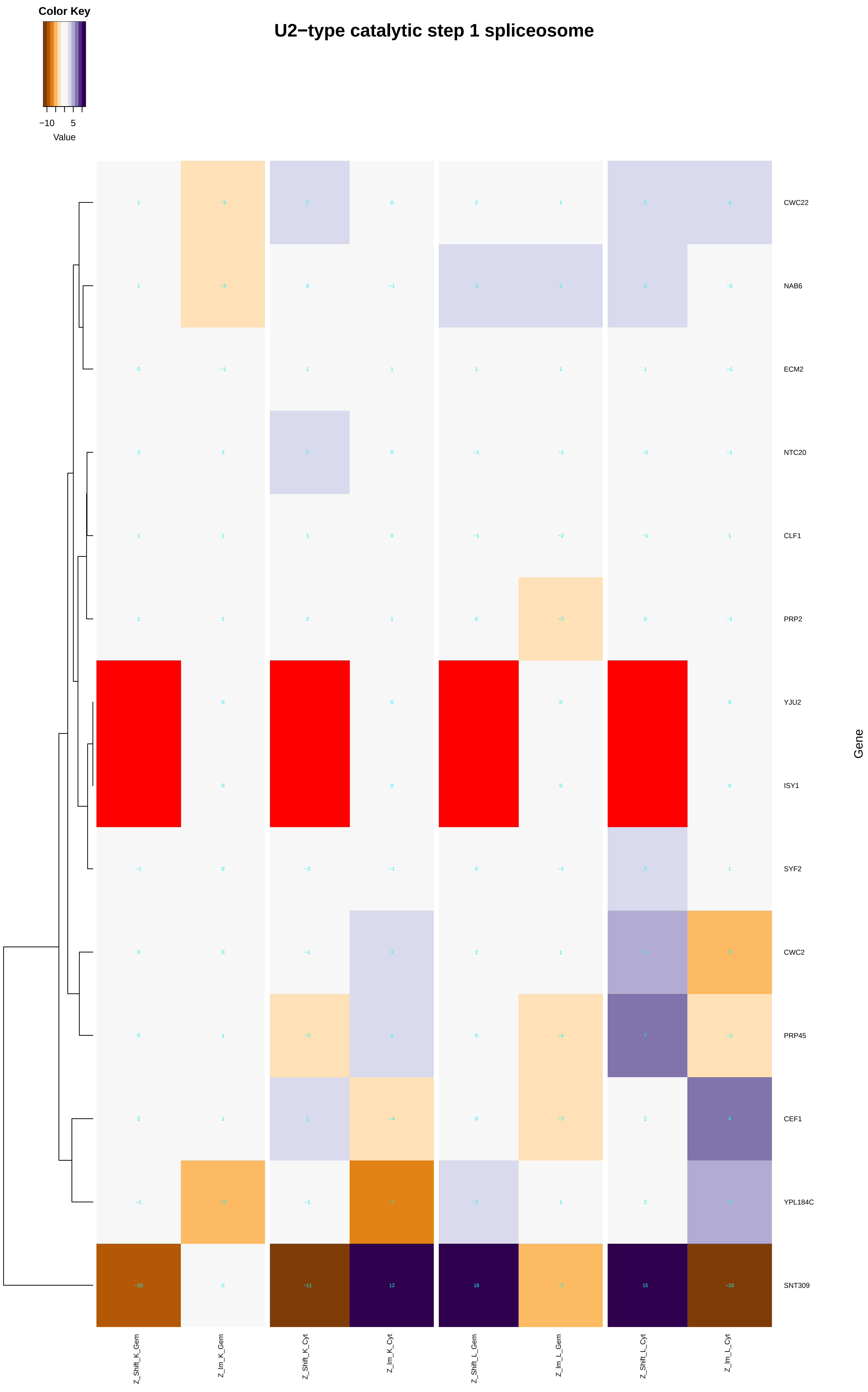

U2−type catalytic step 1 spliceosome
Color Key
−10
5
Value
CWC22
NAB6
ECM2
NTC20
CLF1
PRP2
YJU2
ISY1
SYF2
CWC2
PRP45
CEF1
YPL184C
SNT309
Gene
Z_Shift_K_Gem
Z_Im_K_Gem
Z_Shift_K_Cyt
Z_Im_K_Cyt
Z_Shift_L_Gem
Z_Im_L_Gem
Z_Shift_L_Cyt
Z_Im_L_Cyt

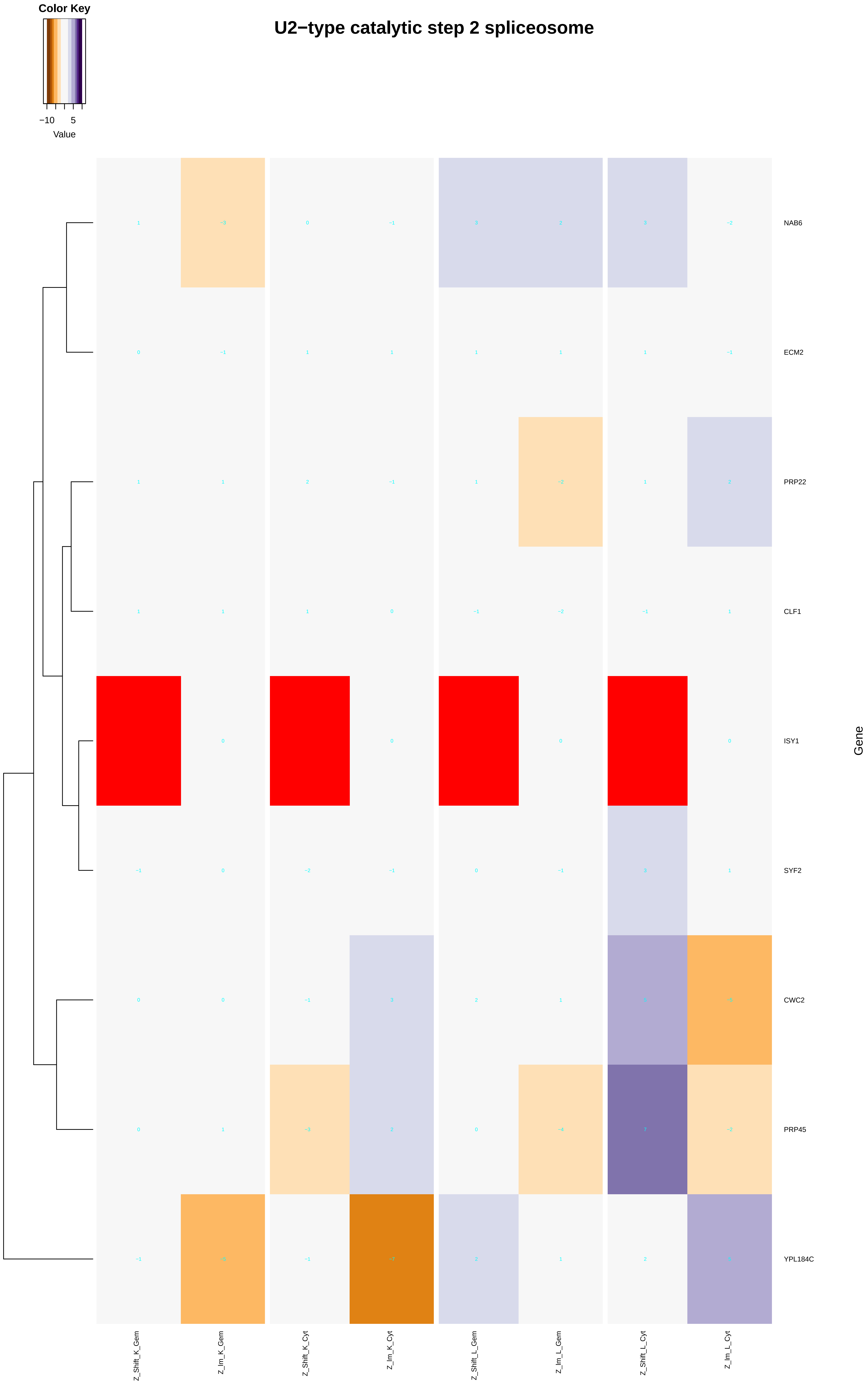
Color Key
−10
5
Value
U2−type catalytic step 2 spliceosome
NAB6
ECM2
PRP22
CLF1
ISY1
SYF2
CWC2
PRP45
YPL184C
Gene
Z_Shift_K_Gem
Z_Im_K_Gem
Z_Shift_K_Cyt
Z_Im_K_Cyt
Z_Shift_L_Gem
Z_Im_L_Gem
Z_Shift_L_Cyt
Z_Im_L_Cyt

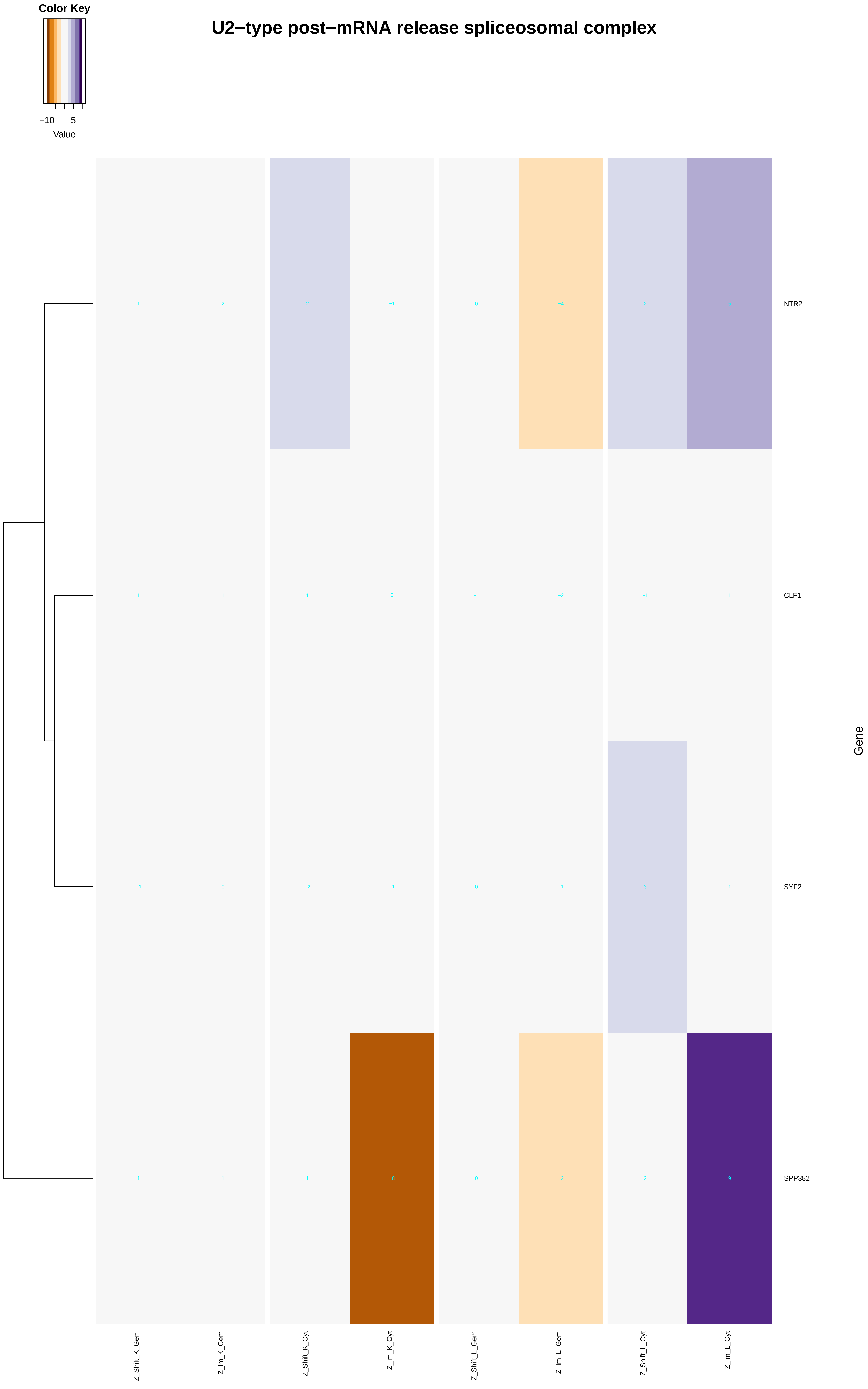

Color Key
−10
5
Value
U2−type post−mRNA release spliceosomal complex
NTR2
CLF1
SYF2
SPP382
Gene
Z_Shift_K_Gem
Z_Im_K_Gem
Z_Shift_K_Cyt
Z_Im_K_Cyt
Z_Shift_L_Gem
Z_Im_L_Gem
Z_Shift_L_Cyt
Z_Im_L_Cyt

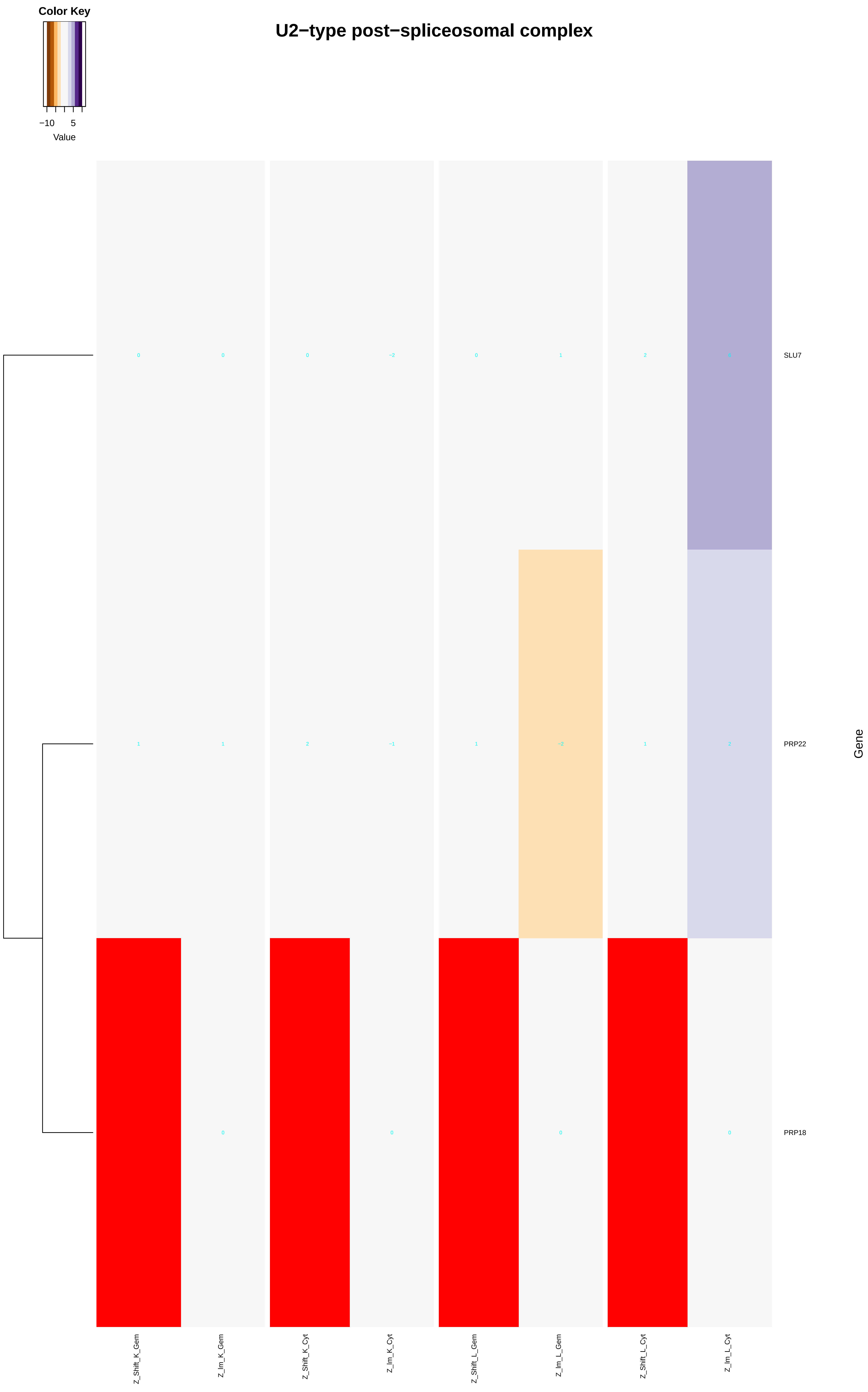
U2−type post−spliceosomal complex
Color Key
−10
5
Value
SLU7
PRP22
PRP18
Gene
Z_Shift_K_Gem
Z_lm_K_Gem
Z_Shift_K_Cyt
Z_lm_K_Cyt
Z_Shift_L_Gem
Z_lm_L_Gem
Z_Shift_L_Cyt
Z_lm_L_Cyt
